# A “Window of Therapeutic Opportunity” for Anti-Cytokine Therapy in Patients With Coronavirus Disease 2019

**DOI:** 10.3389/fimmu.2020.572635

**Published:** 2020-10-06

**Authors:** Ludovico De Stefano, Francesca Bobbio-Pallavicini, Antonio Manzo, Carlomaurizio Montecucco, Serena Bugatti

**Affiliations:** ^1^ Division of Rheumatology, IRCCS Policlinico San Matteo Foundation, Pavia, Italy; ^2^ Department of Internal Medicine and Therapeutics, University of Pavia, Pavia, Italy

**Keywords:** cytokine storm, coronavirus, tocilizumab (IL-6 inhibitor), T cell, immune activation, innate and adaptive immune response

## Abstract

The effects of cytokine inhibition in the different phases of the severe coronavirus disease 2019 (COVID-19) are currently at the center of intense debate, and preliminary results from observational studies and case reports offer conflicting results thus far. The identification of the correct timing of administration of anti-cytokine therapies and other immunosuppressants in COVID-19 should take into account the intricate relationship between the viral burden, the hyperactivation of the innate immune system and the adaptive immune dysfunction. The main challenge for effective administration of anti-cytokine therapy in COVID-19 will be therefore to better define a precise “window of therapeutic opportunity.” Only considering a more specific set of criteria able to integrate information on direct viral damage, the cytokine burden, and the patient’s immune vulnerability, it will be possible to decide, carefully balancing both benefits and risks, the appropriateness of using immunosuppressive drugs even in patients affected primarily by an infectious disease.

## Introduction

The coronavirus disease 2019 (COVID-19), caused by severe acute respiratory syndrome coronavirus 2 (SARS-CoV-2), has a wide spectrum of clinical expressiveness ranging from asymptomatic or paucisymptomatic infection to life-threating multiple organ failure. In most severe forms, SARS-CoV-2 infection leads to fulminant pneumonia and acute respiratory distress syndrome (ARDS) with a mortality rate approaching 40–50% (1). Clinical deterioration generally occurs several days after the onset of symptoms, in association with declining viral titers (2), suggesting that part of pathophysiology may be driven by dysregulated immune responses rather than by direct viral damage. Uncontrolled hyperinflammation has indeed been recognized as a pivotal pathogenetic event in COVID-19 (3), with the release of inflammatory cytokines which are injurious to host cells similarly to what happens in other hyperinflammatory syndromes characterized by cytokine storms, such as the cytokine release syndrome (CRS), secondary hemophagocytic lymphohistiocytosis (sHLH) and macrophage activation syndrome (MAS) (4). The known sensitivity of these syndromes to cytokine-directed therapies has fueled many expectations also for patients with COVID-19. However, the precise nature and role of hyperinflammation in severe COVID-19 remain poorly defined, and the risk-benefit ratio of cytokine inhibition in the different phases of the disease is largely debated (5–8). As a matter of facts, preliminary clinical results on the efficacy of therapeutic blockade of interleukin (IL)-6, IL-1, tumor necrosis factor (TNF), and Janus kinase signaling are thus far mixed (9–23) (**Table 1**), possibly also due to the different timing of drug administration in different reports. Early immunosuppression during an infectious disease indeed incurs risks; on the other hand, however, suppression of pathogenic hyperinflammation may be more effective in the initial stages before clinical deterioration occurs. The identification of the correct timing of administration of immunosuppressive drugs remains therefore a research priority for the personalized management of COVID-19.

**Table 1:** A selection of observational cohort studies on anti-cytokine therapy and immunosuppressants in coronavirus disease 2019 (COVID-19).

	Study design	Country	Intervention	No. of patients	Population	Outcomes
Xu X (9)	Case series	China	Tocilizumab 4–8 mg/kg i.v. Maximum two doses	21	Severe or critical	Clearance of viral load All patients discharged
Sciascia S (10)	Prospective single-arm multicenter study	Italy	Tocilizumab 8 mg/kg i.v. or 324 mg s.c. Two doses in 82.5%	63	SpO2 ≤ 93% or PaO2/FiO2 ≤ 300 CRP > 10 ULN, ferritin > 1,000 ng/ml, D-dimer > 10 ULN, LDH > 2 ULN (at least 3)	Mortality rate 11% Use within 6 days from admission associated with improved survival (HR 2.2, 95% CI 1.3–6.7, p < 0.05)
Toniati P (11)	Prospective single-arm single-center study	Italy	Tocilizumab 8 mg/kg i.v. Two doses (three doses in 13%)	100	Patients requiring ventilatory support 43% requiring mechanical ventilation	Mortality rate 20% Improvement/stabilization of respiratory condition in 77%
SMACORE (12)	Retrospective single-center cohort study	Italy	Tocilizumab 8 mg/kg i.v. Maximum two doses *vs*. standard of care	21 + 21 propensity-score matched	PaO2/FiO2 <300 CRP > 5 mg/dl	No reduction in mortality (OR 0.78, 95% CI 0.06–9.34, p = 0.84) No reduction in ICU admission (OR 0.11, 95% CI 0.00–3.38, p = 0.22)
TOCI-RAF (13)	Retrospective single-center cohort study	Italy	Tocilizumab 400 mg i.v. Two doses in 30% *vs*. standard of care	32 + 33	SpO2 ≤ 92% or PaO2/FiO2 ≤ 300 CRP > 100 mg/l or ferritin > 900 ng/ml, LDH >220 U/l	No reduction in mortality rate (16 *vs*. 33%, p = 0.15) No difference in clinical improvement (69 *vs*. 61%, p = 0.61)
Price CC (14)	Retrospective single-center cohort study	USA	Tocilizumab 8 mg/kg i.v. One dose (two doses admitted) *vs*. standard of care	153 + 86	Patients requiring ≥ 3 L/min of oxygen (mechanical ventilation included)	Reduced survival in severe *vs*. non-severe disease (78 *vs*. 93%, p < 0.001) in the overall cohort similar survival in severe *vs*. non-severe disease with tocilizumab (83 *vs*. 91%, p = 0.11)
Quartuccio L (15)	Retrospective single-center case-control study	Italy	Tocilizumab 8 mg/kg i.v. One dose *vs*. standard of care	42 + 69	Hospitalized	Good outcome in 57.7% of ventilated and 93.7% of non-ventilated patients treated with tocilizumab
Rossotti R (16)	Retrospective single-center cohort study	Italy	Tocilizumab 8 mg/kg i.v. One dose (two doses admitted) *vs*. standard of care	74 + 148	SpO2 ≤ 93% or PaO2/FiO2 ≤ 300 CRP > 1 mg/dl, ferritin > 500 ng/ml, D-dimer > 1.5 mcg/ml, IL-6 > 40 pg/ml (any)	Reduction in mortality (HR 0.499, 95% CI 0.262–0.952, p = 0.035) Longer hospital stay (HR 1.65, 95% CI 1.088–2.524, p = 0.019) due to adverse events
De Rossi N (17)	Retrospective single-center cohort study	Italy	Tocilizumab 324–400 mg s.c. One dose *vs*. standard of care	90 + 68	SpO2 ≤ 93% or PaO2/FiO2 ≤ 300 not requiring mechanical or invasive ventilation	Reduction in mortality rate (7.7 *vs*. 50%, HR 0.57, 95% CI 0.017–0.187, p < 0.001)
Guaraldi G (18)	Retrospective multicenter cohort study	Italy	Tocilizumab 8 mg/kg i.v. or 324 mg s.c. Two doses *vs*. standard of care	179 + 365	RR ≥ 30 breaths/min, SpO2 ≤ 93% or PaO2/FiO2 ≤ 300 + lung infiltrates >50%	Reduction in mortality or risk of invasive mechanical ventilation (HR 0.61, 95% CI 0.40–0.92, p = 0.02)
Biran N (19)	Retrospective multicenter cohort study	USA	Tocilizumab 400 mg i.v. One dose (two doses in 12%) *vs*. standard of care	210 + 420 propensity-score matched	Patients requiring ICU support PaO2/FiO2 <300 ≈95% intubation or ventilator ≈95%	Reduction in mortality rate (49 *vs*. 61%, HR 0.64, 95% CI 0.47–0.87, p = 0.004)
Della-Torre E (20)	Open-label single-center observational study	Italy	Sarilumab 400 mg i.v. One dose *vs*. standard of care	28 + 28	SpO2 ≤ 92% or PaO2/FiO2 ≤ 300 CRP ≥ 100 mg/l, ferritin ≥ 900 ng/ml, IL-6 ≥ 40 pg/ml (any), LDH > ULN	No reduction in mortality (HR 0.36, 95% CI 0.08–1.68, p = 0.21) Longer median time to death (19 days, IQR 13–26 *vs*. 4 days, IQR 3–4, p = 0.006)
Cavalli G (21)	Retrospective single-center cohort study	Italy	Anakinra Low-dose 100 mg s.c. twice daily *vs*. high-dose 5 mg/kg i.v. twice daily *vs*. standard of care	7 + 29 + 16	PaO2/FiO2 ≤ 200 not requiring mechanical ventilation CRP > 100 mg/l or ferritin > 900 ng/ml	Reduction in mortality (HR 0.20, 95% CI 0.04–0.63, p = 0.009) for high-dose anakinra
Ana-COVID (22)	Retrospective single-center cohort study	France	Anakinra 100 mg s.c. twice daily for 72 h, 100 mg daily for 7 days *vs*. standard of care	52 + 44	SpO2 ≤ 93% under 6 L/min of oxygen	Reduction in mortality (HR 0.30, 95% CI 0.12–0.71, p = 0.0063) Reduction in mechanical ventilation (HR 0.22, 95% CI 0.09–0.56, p = 0.0015)
Cantini F (23)	Retrospective multicenter cohort study	Italy	Baricitinib 4 mg/day for 2 weeks *vs*. standard of care	113 + 78	SpO2 > 92% in ambient air and PaO2/FiO2 100–300	Reduction in mortality rate (0 *vs*. 6.4%, p = 0.010) Reduction in ICU admission (0.88 *vs*. 17.9%, p = 0.019)
Fadel R (24)	Retrospective multicenter cohort study	USA	Metylprednisolone max 0.5–1 mg/kg/day in for 7 days *vs*. standard of care	132 + 81	Mild, moderate, and severe	Reduction in mortality rate (13.6 *vs*. 26.3%, p = 0.024) Reduction in mechanical ventilation (21.7 *vs*. 36.6%, p = 0.025)
Li Q (25)	Retrospective single-center cohort study	China	Metylprednisolone max 40 mg/day for 5 days *vs*. standard of care	55 + 55 propensity score-matched	Non-severe COVID-19 pneumonia RR < 30/min SpO2 > 93% in ambient air PaO2/FiO2 >300	No reduction in mortality rate (1.8 *vs*. 0%, p = 0.315) Longer duration of fever (median 5 *vs*. 3 days, p < 0.001) Longer virus clearance time (median 18 *vs*. 11 days, p < 0.001) longer hospital stay (median 23 *vs*. 15 days, p < 0.001)
Keller MJ (26)	Retrospective single-center cohort study	USA	Glucocorticoids *vs*. standard of care	140 + 1,666	Hospitalized	No reduction in mortality (HR 1.20, 95% CI 0.68–2.10) in the overall group No reduction in mechanical ventilation (HR 1.34, 95% CI 0.71–2.52) in the overall group Reduction in mortality or mechanical ventilation (HR 0.20, 95% CI 0.06–0.67) in patients with CRP ≥ 20 mg/dl.

CRP, C-reactive protein; FiO2, fractional inspired oxygen; ICU, intensive care unit; IL, interleukin; LDH, lactate dehydrogenase; Pa02, arterial oxygen partial pressure; RR, respiratory rate; SpO2, peripheral oxygen saturation.

Tocilizumab = anti-IL-6 receptor monoclonal antibody.

Sarilumab = anti-IL-6 receptor monoclonal antibody.

Anakinra = anti-IL-1 receptor antagonist.

Baricitinib = anti-Janus kinase inhibitor-1 and -2.

## The Complex Interplay Between the Viral Burden, Innate, and Adaptive Immune Responses in Coronavirus Disease 2019

The health analytic platform OpenSAFELY established in the United Kingdom has recently confirmed that COVID-19-related deaths are strongly associated with demographic factors and comorbidities such as increasing age, male gender, obesity, cardiovascular and respiratory diseases (27). Risk prediction and tailored treatment solely based on such generic parameters is however largely inaccurate, and COVID-19 outcomes likely depend on a number of other variables influencing host-pathogen interactions, including SARS-CoV-2 genetic variants as well as host genetic susceptibility. Research in this area is only in its infancy. Also building on previous experience with other Coronaviridae, several studies are investigating the link between SARS and genetic variants in innate immune response (28), including mannose‐binding lectin deficiency and polymorphisms, which are involved in the inactivation of a variety of respiratory pathogens through direct binding and complement activation (29). Similarly, adaptive immune dysfunction is appearing as a key, and recent studies have confirmed the role of CD3^+^ T cell cytopenia in determining COVID-19 progression and fatality (30). Discovery of virus and host genomic factors will undoubtedly support risk stratification and targeted treatment; however, as genomic studies require long times before entering clinical practice, it is urgent to integrate easily accessible information on the dynamics and pathogenicity of the immune response during the different phases of SARS-CoV-2 infection.

The relative contribution of viral damage and of innate and adaptive immune responses in course of COVID-19 greatly varies over time (31–33). In the early phases, common to all patients, pathogenic mechanisms are mainly driven by the cytotoxic role directly exerted by SARS-CoV-2, which usually affects only the upper respiratory tract. The early antiviral response mostly relying on type-I interferon (IFN) and natural killer (NK) cells, and the subsequent CD8^+^ T cell-mediated killing of virally infected cells as well as CD4^+^ T cell-dependent antibody production, allow the rapid reduction of the viral load, the paucisymptomatic character of the disease, and its recovery. However, in up to 20% of the patients, virus-induced immunosuppression occurs. This is highlighted by a marked reduction of CD4+ and CD8+ T lymphocytes in peripheral blood, defective CD8+ T cell and NK cell function, low IFN-γ production, and low specific IgG antibodies (34–37). The impairment of the initial antiviral defense mechanisms favors a sustained increase in the viral load capable of conditioning disease progression, especially in organs that have high angiotensin-converting enzyme 2 expression, such as the lungs but also the endothelium, the heart, the kidney, and the intestine (38, 39). The clinical picture can evolve rapidly, with the development of interstitial pneumonia which can progress into ARDS (40). In these early stages, lung damage is mainly driven by SARS-CoV-2 cytotoxicity, while inflammatory responses aim at eliminating the pathogen ultimately leading to tissue repair. Accordingly, longitudinal immune profiling of hospitalized COVID-19 cases with different outcomes has recently shown that, despite similar levels of inflammatory cytokines in the first 10 days from symptom onset, patients with less severe disease evolution also express mediators of wound healing and tissue repair (41). In this phase, the therapeutic strategy should thus include the use of antiviral drugs and of treatments aimed at cautiously enhance immune responses. Conversely, the use of drugs that compromise the efficiency of the immune system could be counterproductive, as patients with high viral loads and long virus-shedding periods are at higher risk of severe COVID-19 (42).

In immunocompromised patients in whom the therapeutic strategies adopted and the presence of comorbidities do not allow adequate control of the viral load, extensive tissue damage, and subsequent uncontrolled inflammatory innate responses with exaggerated myeloid-derived cytokine production can occur (43–46). The clinical picture suddenly and unexpectedly changes with fever, respiratory failure, and ARDS associated with increased levels of acute phase reactants, neutrophilia, thrombocytosis, anemia, signs of coagulopathy, and cell lysis. These acute inflammatory mechanisms may precipitate tissue damage both locally (especially at the lung level) (47) and systemically, thus playing an even greater pathogenetic role than that played by direct viral damage, and significantly affecting mortality. This hyperinflammatory syndrome shares pathophysiological similarities with cytokine storms in CRS, sHLH and MAS, conditions that sometimes complicate viral infections, systemic autoimmune and autoinflammatory diseases, hematologic diseases, and medications such as engineered T cell therapy (48–51). Experience from these conditions has encouraged the use of anti-cytokine therapy also for the management of COVID-19. However, the clinical picture of the cytokine storm in COVID-19, especially when it is not associated with multi-organ damage secondary to coagulopathy, is different from that of the classic sHLH/MAS: it is mostly anatomically compartmentalized to the lungs, not associated with organomegaly and not accompanied by pancytopenia (5). Accordingly, serum levels of IL-6 are lower in COVID-19 compared to other CRS (52). Furthermore, hypercytokinemia should be considered as a general marker of SARS-CoV-2, even in the absence of a cytokine storm, and this makes COVID-19 CRS not easily distinguishable from ARDS pathogenetically correlated only with viral cytotoxic activity, also because levels of inflammatory markers and cytokines not always correlate with outcome (53). Finally, compared to sHLH/MAS, the CRS in COVID-19 occurs in the context of immunodeficiency and immunoparalysis (5, 30, 54), thus imposing caution on further iatrogenic immunosuppression.

## Challenges in the Identification of a “Window of Opportunity” for Immunosuppression in Coronavirus Disease 2019

The identification of the correct timing of administration of anti-cytokine therapies and other immunosuppressants in COVID-19 should thus take into account the intricate relationship between the viral burden, the hyperactivation of the innate immune system, and the adaptive immune dysfunction. Although the therapeutic window in CRS is narrow, and timely control of the cytokine storm is crucial to reduce short-term mortality, premature use of immunosuppressants could indeed further compromise viral shedding with the risk of increasing viral replication and tissue damage directly induced by the virus (43). Furthermore, iatrogenic immunosuppression could promote bacterial, fungus, or viral infectious complications, as shown with the targeting of IL-6 with tocilizumab in observational studies on patients not requiring mechanical ventilation (18, 55). Also at later stages, pharmacologically induced immunosuppression in already immunocompromised patients could adversely affect the course of the disease, as suggested by the finding of significantly longer periods of hospitalization and higher rates of mortality in ventilated patients treated with tocilizumab (15, 16) or high-doses corticosteroids (56, 57).

The main challenge for effective anti-cytokine therapy in COVID-19 will be therefore to better define a precise “window of therapeutic opportunity,” a phase of the disease during which the benefits of cytokine inhibition are prevalent on the inevitable consequences of immunosuppression. Although > 100 randomized clinical trials (RCTs) on different cytokine inhibitors in COVID-19 are underway, the identification of such a “window” remains elusive, and only few studies have tried to integrate further patients’ characterization beyond the clinical status among the inclusion criteria (**Table 2**).

**Table 2:** A selection of the inclusion criteria of some randomized clinical trials on anti-cytokine therapy for coronavirus disease 2019 (COVID-19).

Study	Clinical setting	Respiratory parameters	Signs of hyperinflammation
COVACTA Tocilizumab (USA)	Hospitalized with COVID-19 pneumonia confirmed per WHO criteria and evidenced by chest X-ray or CT scan	SpO2 at rest in ambient air ≤ 93% PaO2/FiO2 < 300 mmHg	
TOCIVID Tocilizumab (Italy)	Virological diagnosis of SARS-CoV-2 infection (real-time PCR) Hospitalized with COVID-19 pneumonia confirmed per WHO criteria and evidenced by chest X-ray or CT scan	SpO2 at rest in ambient air < 93% or Requiring oxygen therapy or Requiring mechanical ventilation (either NIV or IMV)	
TOC-COVID Tocilizumab (Germany)	Virological diagnosis of SARS-CoV-2 infection (real-time PCR) Hospitalized with symptoms	SpO2 at rest in ambient air < 92% or Requiring oxygen therapy > 6 L O2/min or Requiring mechanical ventilation (either NIV or IMV)	
NCT04346355 Tocilizumab (Italy)	Virological diagnosis of SARS-CoV-2 infection (real-time PCR) Hospitalized with COVID-19 pneumonia confirmed per WHO criteria and evidenced by chest X-ray or CT scan or pulmonary ultrasound	PaO2/FiO2 200–300 mmHg	At least one of the following: At least one body temperature measurement >38°C in the past 2 days CRP ≥10 mg/dl CRP increase of at least twice the basal value
ChiCTR2000029765 Tocilizumab (China)	Hospitalized with COVID-19 pneumonia confirmed per WHO criteria: Regular Severe Critical	Case definition: Regular: patients with dual pulmonary lesions accompanied by fever or no fever Severe: any of the following: RR ≥ 30 breaths/min; SpO2 at rest in ambient air ≤ 93%; PaO2/FiO2 ≤ 300 mmHg Critical: any of the following: respiratory failure and mechanical ventilation; shock; organ failure that requires ICU monitoring	Increased IL-6 (ELISA method)
2020COVID-19TCZ Tocilizumab (Belgium)	Hospitalized with COVID-19 pneumonia confirmed per WHO criteria and evidenced by chest X-ray or CT scan	Severe COVID-19 pneumonia (three of the following): Patient wheezing or unable to speak in full sentences while at rest/with minimal effort RR > 22 breaths/min PaO2 < 65 mmHg or SpO2 < 90% Sudden significant chest imaging worsening (despite standard of care which may include antiviral treatment, low dose steroids, and antibiotics)	
NCT04359901 Sarilumab (USA)	Virological diagnosis of SARS-CoV-2 infection (real-time PCR) Moderate COVID-19 disease	Score of 1–3 (out of 3) on a modified Brescia COVID respiratory severity score (BCRSS): Wheezing or inability to speak complete sentences without effort and/or RR > 22 breaths/min and/or SpO2 < 90% on room air or SpO2 < 94% on > 2 l O2/min and/or Any worsening of chest X-ray findings	
NCT04315298 Sarilumab (USA)	Virological diagnosis of SARS-CoV-2 infection (real-time PCR) Hospitalized with COVID-19 pneumonia	Requiring oxygen therapy or Requiring mechanical ventilation (either NIV or IMV)	
NCT04324021 Anakinra (Italy)	Diagnosis of SARS-Cov-2 infection as per hospital routine	PaO2/FiO2 200–300 mmHg or RR ≥ 30 breaths/min or SpO2 at rest in ambient air < 93%	Lymphocyte counts < 1,000 cells/microl and at least two of the following: Ferritin > 500 ng/ml LDH > 300 U/l D-Dimers > 1,000 ng/ml
COV-BARRIER Baricitinib (USA)	Virological diagnosis of SARS-CoV-2 infection (real-time PCR) COVID-19 pneumonia confirmed per WHO criteria and evidenced by radiological findings or Active COVID-19 infection	SpO2 at rest in ambient air <94% or PaO2/FiO2 < 300 mmHg or Active infection: Fever and/or Vomiting and/or diarrhea and/or dry coug and/or RR > 24 breaths/min	At least one of the following > 1 ULN: CRP D-Dimer LDH Ferritin
NCT04358614 Baricitinib (Italy)	Virological diagnosis of SARS-CoV-2 infection (real-time PCR) Hospitalized with COVID-19 pneumonia confirmed per WHO criteria and evidenced by chest X-ray or CT scan or pulmonary ultrasound	SpO2 at rest in ambient air > 92% PaO2/FiO2 > 100–300 mmHg	At least three of the following: Fever Cough Myalgia Fatigue
COMBAT-19 Mavrilimumab (Italy)	Virological diagnosis of SARS-CoV-2 infection (real-time PCR) Hospitalized with COVID-19 pneumonia confirmed per WHO criteria and evidenced by chest X-ray or CT scan	SpO2 at rest in ambient air ≤ 92% PaO2/FiO2 ≤ 300 mmHg	LDH > 1 ULN and at least one of the following: Fever >38°C CRP ≥ 60 mg/l Ferritin ≥ 1,000 microg/l

COVID-19, coronavirus disease 2019; CRP, C-reactive protein; CT, computed tomography; FiO2, fractional inspired oxygen; IL, interleukin; IMV, invasive mechanical ventilation; LDH, lactate dehydrogenase; NIV, noninvasive ventilation; Pa02, arterial oxygen partial pressure; PCR, polymerase chain reaction; RR, respiratory rate; SARS-CoV-2, severe acute respiratory syndrome coronavirus 2; SpO2, peripheral oxygen saturation; ULN, upper limit of normal; WHO, World Health Organization.

Typical chest imaging findings suggestive of COVID-19 according to the WHO (https://apps.who.int/iris/bitstream/handle/10665/333912/WHO-2019-nCoV-Surveillance_Case_Definition-2020.1-eng.pdf?sequence=1&isAllowed=y).

•Chest radiography: hazy opacities, often rounded in morphology, with peripheral and lower lung distribution.

•Chest CT: multiple bilateral ground glass opacities, often rounded in morphology, with peripheral and lower lung distribution.

•Lung ultrasound: thickened pleural lines, B lines (multifocal, discrete, or confluent), consolidative patterns with or without air bronchograms.

Tocilizumab = anti-IL-6 receptor monoclonal antibody.

Sarilumab = anti-IL-6 receptor monoclonal antibody.

Anakinra = anti-IL-1 receptor antagonist.

Baricitinib = anti-Janus kinase inhibitor-1 and -2.

Mavrilimumab = anti-granulocyte-macrophage colony-stimulating factor receptor monoclonal antibody.

Most of the protocols essentially require, among the inclusion criteria, the presence of pneumonia not otherwise defined if not by the extent of functional impairment, with a tendency to focus mainly on the early stages of the disease, in the absence of ARDS or systemic involvement. Some observational studies continue to suggest that immunosuppressants such as glucocorticoids and IL-6 antagonists (alone or in combination) are advantageous if administered early at hospital admission (17, 24, 58) (**Table 1**). However, the RECOVERY trial has clearly shown that benefits from dexamethasone are restricted to those patients with at least 7 days of symptoms and those requiring invasive or non-invasive ventilation (59), suggesting that, based on the clinical criterion alone, only a late phase of COVID-19 is dominated by pathogenic immunity. Accordingly, the use of early short-term corticosteroid therapy, even at low-doses, has been associated with worse clinical outcomes in non-severe COVID-19 pneumonia in observational studies (25) (**Table 1**). In line with these findings, following preliminary results, RCTs on sarilumab (another IL-6 receptor antagonist) have been amended to enroll only critical patients (https://investor.regeneron.com/news-releases/news-release-details/regeneron-and-sanofi-provide-update-us-phase-23-adaptive), and the COVACTA trial on tocilizumab recently ended recruitment because neither primary nor secondary end-points were met (https://www.roche.com/media/releases/med-cor-2020-07-29.htm).

Better and still clinically feasible characterization of those patients with an hyperinflammatory syndrome potentially susceptible to immunosuppression could derive from the integration of markers of inflammation, tissue damage, cell lysis, and coagulopathy. This has indeed been the rationale for the development of the H-score for the diagnosis of sHLH and MAS, which includes, among the others, hyperferritinemia, cytopenias, and liver damage (60). However, the many clinical and pathogenetic differences between COVID-19 hyperinflammation and sLHL/MAS limit the generalizability of the H-score to COVID-19 associated cytokine storm, and only few patients with severe COVID-19 achieve diagnostic cut-offs of >169, mainly due to lower ferritin levels, absence of pancytopenia and hypofibrinogenemia (61). Similarly, the prevalence of the hyperinflammatory phenotype in ARDS due to COVID-19 is lower compared to that observed in non-COVID-19 ARDS despite its higher mortality (53). However, laboratory indicators of hyperinflammation may still be of value in the identification of those COVID-19 patients more at risk of escalation of respiratory support and death. Using cut-off values of C-reactive protein (CRP) concentrations greater than 150 mg/L or ferritin concentrations greater than 1,500 μg/L, Manson JJ and colleagues (62) recently described an hyperinflammatory phenotype of COVID-19 characterized by poor clinical outcomes irrespective of other demographic and clinical variables. Patients’ stratification based on this approach could be of promise. Accordingly, in an observational study on hospitalized COVID-19 cases, early use of glucocorticoids was effective only in patients with high CRP levels, being instead associated with increased mortality in case of low CRP (26).

Elevation of inflammatory markers such as CRP, ferritin, and others is however non-specific for cytokine storms. Cytokine assays are ready available in the clinic and testing for serum levels of cytokines known to be involved in pathogenic inflammation could help guiding the rational use of targeted immunomodulatory therapeutic strategies. Accordingly, IL-6 and other pro-inflammatory cytokines have been shown to be predictive of patient outcomes in terms of both disease severity and survival, and integrated models taking into account demographics, comorbidities, markers of inflammation and tissue damage, and cytokines show good performance with areas under the receiver operating characteristic curve approaching 0.8 (63). Several limitations however still exist even using similar approaches. Apart from IL-6, the trend of other cytokines whose targeting is already available in the clinic does not appear uniform in COVID-19 (30, 41, 54, 63), hampering the possibility of tailored treatments and contrasting with the apparent efficacy of some agents, such as IL-1 antagonists, in preliminary observational studies (21, 22). Even in the case of IL-6, no established cut-offs exist. Furthermore, the release of inflammatory cytokines is also part of a well-conserved innate immune response necessary for efficient clearance of infectious agents. Accordingly, levels of IL-6 are similarly increased in the hyperinflammatory phenotype of non-COVID-19 ARDS irrespective of its causative mechanism, but interventions targeting single cytokines in this setting have a long history of failure (64).

Collectively, current clinical, laboratory, and even biological parameters thus do not appear optimal at distinguishing an appropriate from a dysregulated inflammatory response in the context of COVID-19. Ideally, in light of the very pulmonary-centric character of this condition, better knowledge could arise from the analysis of parameters deriving from bronchoalveolar lavage fluid and microscopic examination of transbronchial lung biopsies (**Table 3**). These include cytological analyses with prevalence of granulocytes, macrophages, and lymphocytes; pro-inflammatory cytokine and chemokine secretion including neutrophil recruiting mediators and other attractants of monocytes and immune cells; lung infiltration by monocytes, macrophages, and neutrophils; thrombosis (65–68). The clinical, radiological, and laboratory characteristics already identified in patients with COVID-19 would thus be put in the context of more specific histological, cytological, and immune-inflammatory criteria in order to identify those parameters more accurately signaling the presence of a predominantly immuno-inflammatory pathogenesis which might benefit from immunosuppression (**Table 3**). The evidence of ongoing SARS-CoV-2 replication late in disease would in any case support the associated use of antiviral therapy, even at a point when immunopathology is dominant (69).

**Table 3:** Possible inclusion criteria for anti-cytokine therapy in coronavirus disease 2019 (COVID-19).

Mandatory criteria	
Functional and radiological lung involvement	Hospitalized with COVID-19 pneumonia confirmed per WHO criteria and evidenced by chest X-ray or CT scan SpO2 at rest in ambient air < 90%; PaO2/FiO2 < 300 mmHg
***And***	
Clinical and laboratory signs of exaggerated inflammatory response	At least two of the following criteria: Serum CRP ≥ 15 mg/dl Ferritin > 500 ng/ml LDH > 300 U/l D-Dimer > 1,000 mg/ml Leucocyte count > 10,000/mm^3^ Increase of at least 2 fold of each parameter in serial controls
**Optional criteria**	
Bronchoalveolar lavage fluid analysis	At least one of the following criteria: Prevalence of granulocytes (>5%) and macrophages (>85%) on lymphocytes (<5%) in cytological analysis Increase of pro-inflammatory cytokines including IL-8, IL-6, and IL-1β and neutrophil recruiting mediators and other attractants of monocytes as CXCL1, CXCL2, CXCL3, CXCL5
***Or***	
Transbronchial lung biopsy	At least one of the following criteria: Lung infiltration by monocytes, macrophages and neutrophils, particularly CD163-expressing macrophages in contrast with lower amounts of lymphocytes Diffuse alveolar damage with the formation of hyaline membranes Diffuse thickening of the alveolar wall

CD, cluster of differentiation; COVID-19, coronavirus disease 2019; CRP, C-reactive protein; CT, computed tomography; CXCL, chemokine ligand family; FiO2, fractional inspired oxygen; IL, interleukin; LDH, lactate dehydrogenase; SpO2, peripheral oxygen saturation; WHO, World Health Organization.

In conclusion, despite the emergency situation imposes speed in the identification of the therapeutic options for patients with COVID-19, better harmonization of inclusion criteria and patient stratifications for studies on immunomodulatory therapies should remain a priority. Only considering a more specific set of clinical and pathological criteria, together with the extent of the viral load present in the alveolar bronchial lavage fluid and parameters useful to quantify the patient’s immune vulnerability (lymphocyte count, CD4+ and CD8+ T cell levels, markers of lymphocyte exhaustion, development of specific antibodies), it will be possible to decide, carefully balancing both benefits and risks, the appropriateness of using immunosuppressive drugs even in patients affected primarily by an infectious disease.

## Data Availability Statement

The original contributions presented in the study are included in the article/supplementary material; further inquiries can be directed to the corresponding author.

## Author Contributions

All the authors contributed to the conception of the commentary. LS drafted the manuscript. All authors contributed to the article and approved the submitted version.

## Funding

This article was supported in part by fundings from the IRCCS Policlinico San Matteo Foundation, Pavia, Italy.

## Conflict of Interest

The authors declare that the research was conducted in the absence of any commercial or financial relationships that could be construed as a potential conflict of interest.

The reviewer LQ declared a past co-authorship with two of the authors CM and SB to the handling editor.

## References

[1] Wu C Chen X Cai Y Xia J Zhou X Xu S Risk Factors Associated With Acute Respiratory Distress Syndrome and Death in Patients With Coronavirus Disease 2019 Pneumonia in Wuhan, China. JAMA Intern Med (2020) 180:1–11. 10.1001/jamainternmed.2020.0994 PMC7070509 32167524

[2] Joynt GM Wu WK Understanding COVID-19: what does viral RNA load really mean? Lancet Infect Dis (2020) 20:635–6. 10.1016/S1473-3099(20)30237-1 PMC7118539 32224308

[3] Merad M Martin JC Pathological inflammation in patients with COVID-19: a key role for monocytes and macrophages. Nat Rev Immunol (2020) 20:355–62. 10.1038/s41577-020-0331-4 PMC7201395 32376901

[4] Behrens EM Koretzky GA Review: cytokine storm syndrome: looking toward the precision medicine era. Arthritis Rheumatol (2017) 69:1135–43. 10.1002/art.40071 28217930

[5] McGonagle D Sharif K O’Regan A Bridgewood C The Role of Cytokines including Interleukin-6 in COVID-19 induced Pneumonia and Macrophage Activation Syndrome-Like Disease. Autoimmun Rev (2020) 19:102537. 10.1016/j.autrev.2020.102537 32251717 PMC7195002

[6] Jamilloux Y Henry T Belot A Viel S Fauter M El Jammal T Should we stimulate or suppress immune responses in COVID-19? Cytokine and anti-cytokine interventions. Autoimmun Rev (2020) 19:102567. 10.1016/j.autrev.2020.102567 32376392 PMC7196557

[7] Alijotas-Reig J Esteve-Valverde E Belizna C Selva-O’Callaghan A Pardos-Gea J Quintana A Immunomodulatory Therapy for the Management of Severe COVID-19. Beyond the Anti-Viral Therapy: A Comprehensive Review. Autoimmun Rev (2020) 19:102569. 10.1016/j.autrev.2020.102569 32376394 PMC7252146

[8] Ruscitti P Berardicurti O Iagnocco A Giacomelli R Cytokine storm syndrome in severe COVID-19. Autoimmun Rev (2020) 19:102562. 10.1016/j.autrev.2020.102562 32376400 PMC7252135

[9] Xu X Han M Li T Sun W Wang D Fu B Effective treatment of severe COVID-19 patients with tocilizumab. Proc Natl Acad Sci U S A (2020) 117:10970–5. 10.1073/pnas.2005615117 PMC7245089 32350134

[10] Sciascia S Aprà F Baffa A Baldovino S Boaro D Boero R Pilot prospective open, single-arm multicentre study on off-label use of tocilizumab in patients with severe COVID-19. Clin Exp Rheumatol (2020) 38:529–32. 32359035

[11] Toniati P Piva S Cattalini M Garrafa E Regola F Castelli F Tocilizumab for the treatment of severe COVID-19 pneumonia with hyperinflammatory syndrome and acute respiratory failure: A single center study of 100 patients in Brescia, Italy. Autoimmun Rev (2020) 19:102568. 10.1016/j.autrev.2020.102568 32376398 PMC7252115

[12] Colaneri M Bogliolo L Valsecchi P Sacchi P Zuccaro V Brandolino F Tocilizumab for Treatment of Severe COVID-19 Patients: Preliminary Results from SMAtteo COvid19 REgistry (SMACORE). Microorganisms (2020) 8:695. 10.3390/microorganisms8050695 PMC7285503 32397399

[13] Campochiaro C Della-Torre E Cavalli G De Luca G Ripa M Boffini N Efficacy and safety of tocilizumab in severe COVID-19 patients: a single-centre retrospective cohort study. Eur J Intern Med (2020) 76:43–9. 10.1016/j.ejim.2020.05.021 PMC7242960 32482597

[14] Price CC Altice FL Shyr Y Koff A Pischel L Goshua G Tocilizumab Treatment for Cytokine Release Syndrome in Hospitalized COVID-19 Patients: Survival and Clinical Outcomes. Chest (2020) S0012-3692(20):31670–6. 10.1016/j.chest.2020.06.006 PMC7831876 32553536

[15] Quartuccio L Sonaglia A McGonagle D Fabris M Peghin M Pecori D Profiling COVID-19 pneumonia progressing into the cytokine storm syndrome: Results from a single Italian Centre study on tocilizumab versus standard of care. J Clin Virol (2020) 129:104444. 10.1016/j.jcv.2020.104444 32570043 PMC7227535

[16] Rossotti R Travi G Ughi N Corradin M Baiguera C Fumagalli R Safety and efficacy of anti-il6-receptor tocilizumab use in severe and critical patients affected by coronavirus disease 2019: A comparative analysis. J Infect (2020) S0163-4453(20):30467–9. 10.1016/j.jinf.2020.07.008 PMC7345400 32652164

[17] De Rossi N Scarpazza C Filippini C Cordioli C Rasia S Mancinelli CR Early use of low dose tocilizumab in patients with COVID-19: A retrospective cohort study with a complete follow-up. EClinicalMedicine (2020) 17:100459. 10.1016/j.eclinm.2020.100459 PMC7366117 32838235

[18] Guaraldi G Meschiari M Cozzi-Lepri A Milic J Tonelli R Menozzi M Tocilizumab in patients with severe COVID-19: a retrospective cohort study. Lancet Rheumatol (2020) 2:e474–84. 10.1016/S2665-9913(20)30173-9 PMC7314456 32835257

[19] Biran N Ip A Ahn J Go RC Wang S Mathura S Tocilizumab among patients with COVID-19 in the intensive care unit: a multicentre observational study. Lancet Rheumatol (2020) 2(10):e603–12. 10.1016/S2665-9913(20)30277-0 32838323 PMC7428303

[20] Della-Torre E Campochiaro C Cavalli G De Luca G Napolitano A La Marca S Interleukin-6 blockade with sarilumab in severe COVID-19 pneumonia with systemic hyperinflammation: an open-label cohort study. Ann Rheum Dis (2020) 3:annrheumdis–2020-218122. 10.1136/annrheumdis-2020-218122 PMC7509526 32620597

[21] Cavalli G De Luca G Campochiaro C Della-Torre E Ripa M Canetti D Interleukin-1 blockade with high-dose anakinra in patients with COVID-19, acute respiratory distress syndrome, and hyperinflammation: a retrospective cohort study. Lancet Rheumatol (2020) 2:e325–31. 10.1016/S2665-9913(20)30127-2 PMC7252085 32501454

[22] Huet T Beaussier H Voisin O Jouveshomme S Dauriat G Lazareth I Anakinra for severe forms of COVID-19: a cohort study. Lancet Rheumatol (2020) 2:e393–400. 10.1016/S2665-9913(20)30164-8 PMC7259909 32835245

[23] Cantini F Niccoli L Nannini C Matarrese D Natale MED Lotti P Beneficial impact of Baricitinib in COVID-19 moderate pneumonia; multicentre study. J Infect (2020) S0163-4453(20):30433–3. 10.1016/j.jinf.2020.06.052 PMC7313480 32592703

[24] Fadel R Morrison AR Vahia A Smith ZR Chaudhry Z Bhargava P Early Short Course Corticosteroids in Hospitalized Patients with COVID-19. Clin Infect Dis (2020) 19:ciaa601. 10.1093/cid/ciaa601 PMC7314133 32427279

[25] Li Q Li W Jin Y Xu W Huang C Li L Efficacy Evaluation of Early, Low-Dose, Short-Term Corticosteroids in Adults Hospitalized with Non-Severe COVID-19 Pneumonia: A Retrospective Cohort Study. Infect Dis Ther (2020) 2:1–14. 10.1007/s40121-020-00332-3 PMC7467137 32880102

[26] Keller MJ Kitsis EA Arora S Chen JT Agarwal S Ross MJ Effect of Systemic Glucocorticoids on Mortality or Mechanical Ventilation in Patients With COVID-19. J Hosp Med (2020) 15:489–93. 10.12788/jhm.3497 PMC7518134 32804611

[27] Williamson EJ Walker AJ Bhaskaran K Bacon S Bates C Morton CE Factors associated with COVID-19-related death using OpenSAFELY. Nature (2020) 584:430–6. 10.1038/s41586-020-2521-4 PMC7611074 32640463

[28] Ovsyannikova IG Haralambieva IH Crooke SN Poland GA Kennedy RB The role of host genetics in the immune response to SARS-CoV-2 and COVID-19 susceptibility and severity. Immunol Rev (2020) 296:205–19. 10.1111/imr.12897 PMC7404857 32658335

[29] Ip WK Chan KH Law HK Tso GHW Kong EKP Wong WHS Mannose-binding lectin in severe acute respiratory syndrome coronavirus infection. J Infect Dis (2005) 191:1697–704. 10.1086/429631 PMC7199483 15838797

[30] Zhang X Tan Y Ling Y Lu F Yi Z Jia X Viral and host factors related to the clinical outcome of COVID-19. Nature (2020) 583:437–00. 10.1038/s41586-020-2355-0 32434211

[31] Ling L Lianfeng L Wei C Taisheng L Hypothesis for potential pathogenesis of SARSCoV-2 infection -a review of immune changes in patients with viral pneumonia. Emerg Microbes Infect (2020) 9:727–32. 10.1080/22221751.2020.1746199 PMC7170333 32196410

[32] Cao X COVID-19: immunopathology and its implications for therapy. Nat Rev Immunol (2020) 9:1–2. 10.1038/s41577-020-0308-3 PMC7143200 32273594

[33] Vardhana SA Wolchok JD The Many Faces of the anti-COVID Immune Response. J Exp Med (2020) 217:e20200678. 10.1084/jem.20200678 32353870 PMC7191310

[34] Chen G Wu D Guo W Cao Y Huang D Wang H Clinical and immunologic features in severe and moderate forms of Coronavirus Disease 2019. J Clin Invest (2020) 130:2620–9. 10.1172/JCI137244 PMC7190990 32217835

[35] Wang X Xu W Hu G Xia S Sun Z Liu Z SARS-CoV-2 infects T lymphocytes through its spike protein-mediated membrane fusion. Cell Mol Immunol (2020) 7:1–3. 10.1038/s41423-020-0424-9 PMC7348563 32651469

[36] Zheng M Gao Y Wang G Song G Liu S Sun D Functional exhaustion of antiviral lymphocytes in COVID-19 patients. Cell Mol Immunol (2020) 17:533–5. 10.1038/s41423-020-0402-2 PMC7091858 32203188

[37] Channappanavar R Fehr AR Vijay R Mack M Zhao J Meyerholz DK Dysregulated type I interferon and inflammatory monocyte-macrophage responses cause lethal pneumonia in SARS-CoV-infected mice. Cell Host Microbe (2016) 19:181–93. 10.1016/j.chom.2016.01.007 PMC4752723 26867177

[38] Hamming I Timens W Bulthuis ML Lely AT Navis G van Goor H Tissue distribution of ACE2 protein, the functional receptor for SARS coronavirus. A first step in understanding SARS pathogenesis. J Pathol (2004) 203:631–7. 10.1002/path.1570 PMC7167720 15141377

[39] Rivellese F Prediletto E ACE2 at the centre of COVID-19 from paucisymptomatic infections to severe pneumonia. Autoimmun Rev (2020) 19:102536. 10.1016/j.autrev.2020.102536 32251718 PMC7195011

[40] Huang C Wang Y Li X Ren L Zhao J Hu Y Clinical features of patients infected with 2019 novel coronavirus in Wuhan, China. Lancet (2020) 395:497–506. 10.1016/S0140-6736(20)30183-5 31986264 PMC7159299

[41] Lucas C Wong P Klein J Castro TBR Silva J Sundaram M Longitudinal analyses reveal immunological misfiring in severe COVID-19. Nature (2020) 584:463–9. 10.1038/s41586-020-2588-y PMC7477538 32717743

[42] Chen W Lan Y Yuan X Deng X Li Y Cai X Detectable 2019-nCoV viral RNA in blood is a strong indicator for the further clinical severity. Emerg Microbes Infect (2020) 9:469–73. 10.1080/22221751.2020.1732837 PMC7054964 32102625

[43] Liu Y Yan LM Wan L Xiang TX Le A Liu JM Viral dynamics in mild and severe cases of COVID-19. Lancet Infect Dis (2020) 20:656–7. 10.1016/S1473-3099(20)30232-2 PMC7158902 32199493

[44] Velazquez-Salinas L Verdugo-Rodriguez A Rodriguez LL Borca MV The role of interleukin 6 during viral infections. Front Microbiol (2019) 10:1057. 10.3389/fmicb.2019.01057 31134045 PMC6524401

[45] Yap JKY Moriyama M Iwasaki A Inflammasomes and Pyroptosis as Therapeutic Targets for COVID-19. J Immunol (2020) 205:307–12. 10.4049/jimmunol.2000513 PMC7343621 32493814

[46] McKechnie JL Blish CA The Innate Immune System: Fighting on the Front Lines or Fanning the Flames of COVID-19? Cell Host Microbe (2020) S1931-3128(20):30291–2. 10.1016/j.chom.2020.05.009 PMC7237895 32464098

[47] Hogner K Wolff T Pleschka S Plog S Gruber AD Kalinke U Macrophage expressed IFN-β contributes to apoptotic alveolar epithelial cell injury in severe influenza virus pneumonia. PloS Pathog (2013) 9:e1003188. 10.1371/journal.ppat.1003188 23468627 PMC3585175

[48] Al-Samkari H Berliner N Hemophagocytic Lymphohistiocytosis. Annu Rev Pathol (2018) 13:27–49. 10.1146/annurev-pathol-020117-043625 28934563

[49] Ravelli A Davì S Minoia F Martini A Cron RQ Macrophage Activation Syndrome. Hematol Oncol Clin North Am (2015) 29:927–41. 10.1016/j.hoc.2015.06.010 26461152

[50] Huang KJ Su IJ Theron M Wu YC Lai SK Liu CC An Interferon-Gamma-Related Cytokine Storm in SARS Patients. J Med Virol (2005) 75:185–94. 10.1002/jmv.20255 PMC7166886 15602737

[51] Maude SL Barrett D Teachey DT Grupp SA Managing cytokine release syndrome associated with novel T cell-engaging therapies. Cancer J (2014) 20:119–22. 10.1097/PPO.0000000000000035 PMC4119809 24667956

[52] Maude S Barrett DM Current status of chimeric antigen receptor therapy for haematological malignancies. Br J Haematol (2016) 172:11–22. 10.1111/bjh.13792 26560054

[53] Sinha P Calfee CS Cherian S Brealey D Cutler S King C Prevalence of phenotypes of acute respiratory distress syndrome in critically ill patients with COVID-19: a prospective observational study. Lancet Respir Med (2020) 27S2213-2600(20)30366-0. 10.1016/S2213-2600(20)30366-0 PMC7718296 32861275

[54] Laing AG Lorenc A Del Molino Del Barrio I Das A Fish M Monin L A dynamic COVID-19 immune signature includes associations with poor prognosis. Nat Med (2020) 10.1038/s41591-020-1038-6 32807934

[55] Antinori S Bonazzetti C Gubertini G Capetti A Pagani C Morena V Tocilizumab for cytokine storm syndrome in COVID-19 pneumonia: an increased risk for candidemia? Autoimmun Rev (2020) 19:102564. 10.1016/j.autrev.2020.102564 32376396 PMC7200127

[56] Russell CD Millar JE Baillie JK Clinical evidence does not support corticosteroid treatment for 2019-nCoV lung injury. Lancet (2020) 395:473–5. 10.1016/S0140-6736(20)30317-2 PMC7134694 32043983

[57] Yang Z Liu J Zhou Y Zhao X Zhao Q Liu J The effect of corticosteroid treatment on patients with coronavirus infection: a systematic review and meta-analysis. J Infect (2020) S0163-4453(20):30191–2. 10.1016/j.jinf.2020.03.062 PMC7195158 32283144

[58] Martínez-Urbistondo D Costa Segovia R Suárez Del Villar Carrero R Risco Risco C Villares Fernández P Early combination of Tocilizumab and Corticosteroids: An upgrade in anti-inflammatory therapy for severe COVID. Clin Infect Dis (2020) 4:ciaa910. 10.1093/cid/ciaa910 PMC7543327 32621739

[59] Brightling C Ustianowski A Elmahi E Prudon B Green C Felton T Dexamethasone in Hospitalized Patients with Covid-19 - Preliminary Report. N Engl J Med (2020) 17:NEJMoa2021436. 10.1056/NEJMoa2021436

[60] Fardet L Galicier L Lambotte O Marzac C Aumont C Chahwan D Development and validation of the HScore, a score for the diagnosis of reactive hemophagocytic syndrome. Arthritis Rheumatol (2014) 66:2613–20. 10.1002/art.38690 24782338

[61] Ruscitti P Bruno F Berardicurti O Acanfora C Pavlych V Palumbo P Lung involvement in macrophage activation syndrome and severe COVID-19: results from a cross-sectional study to assess clinical, laboratory and artificial intelligence-radiological differences. Ann Rheum Dis (2020) 79:1152–5. 10.1136/annrheumdis-2020-218048 PMC7456556 32719039

[62] Manson JJ Crooks C Naja M Ledlie A Goulden B Liddle T COVID-19-associated hyperinflammation and escalation of patient care: a retrospective longitudinal cohort study. Lancet Rheumatol (2020) 2:e594–e602. 10.1016/S2665-9913(20)30275-7 32864628 PMC7442426

[63] Del Valle DM Kim-Schulze S Huang HH Beckmann ND Nirenberg S Wang B An inflammatory cytokine signature predicts COVID-19 severity and survival. Nat Med (2020) 10.1038/s41591-020-1051-9 PMC7869028 32839624

[64] Sinha P Calfee CS Phenotypes in acute respiratory distress syndrome: moving towards precision medicine. Curr Opin Crit Care (2019) 25:12–20. 10.1097/MCC.0000000000000571 30531367 PMC6814152

[65] Xiong Y Liu Y Cao L Wang D Guo M Jiang A Transcriptomic characteristics of bronchoalveolar lavage fluid and peripheral blood mononuclear cells in COVID-19 patients. Emerg Microbes Infect (2020) 9:761–70. 10.1080/22221751.2020.1747363 PMC7170362 32228226

[66] Zhou Z Ren L Zhang L Zhong J Xiao Y Jia Z Heightened innate immune responses in the respiratory tract of COVID-19 patients. Cell Host Microbe (2020) 27:883–90. 10.1016/j.chom.2020.04.017 PMC7196896 32407669

[67] Liao M Liu Y Yuan J Wen Y Xu G Zhao J Single-cell landscape of bronchoalveolar immune cells in patients with COVID-19. Nat Med (2020) 26:842–4. 10.1038/s41591-020-0901-9 32398875

[68] Xu Z Shi L Wang Y Zhang J Huang L Zhang C Pathological findings of COVID-19 associated with acute respiratory distress syndrome. Lancet Respir Med (2020) 8:420–2. 10.1016/S2213-2600(20)30076-X PMC7164771 32085846

[69] Hanley B Naresh KN Roufosse C Nicholson AG Weir J Cooke GS Histopathological findings and viral tropism in UK patients with severe fatal COVID-19: a post-mortem study. Lancet Microbe (2020) 10.1016/S2666-5247(20)30115-4 PMC7440861 32844161

